# Supplementary material for: BrainPhys neuronal medium optimized for imaging and optogenetics in vitro
Source: Nat Commun. 2020 Nov 3;11:5550. doi: 10.1038/s41467-020-19275-x (PMC7642238; doi:10.1038/s41467-020-19275-x)
Supplement: Supplementary file 1 — Supplementary Information [file 41467_2020_19275_MOESM1_ESM.pdf]

BrainPhys neuronal medium optimized for imaging and optogenetics in vitro

Michael Zabolocki, Kasandra McCormack, Mark van den Hurk, Bridget Milky, Andrew Shoubridge, Robert Adams, Jenne Tran, Anita Mahadevan-Jansen, Philipp Reineck, Jacob Thomas, Mark R. Hutchinson, Carmen Mak, Adam Añonuevo, Leon Harold Chew, Adam J. Hirst, Vivian M. Lee, Erin Knock, Cedric Bardy

Supplementary Information

Supplementary Figure 1 (related to Figure 1 and 2). **Influence of BrainPhys Imaging on autofluorescence and signal-to-background ratio compared to competitive media.**

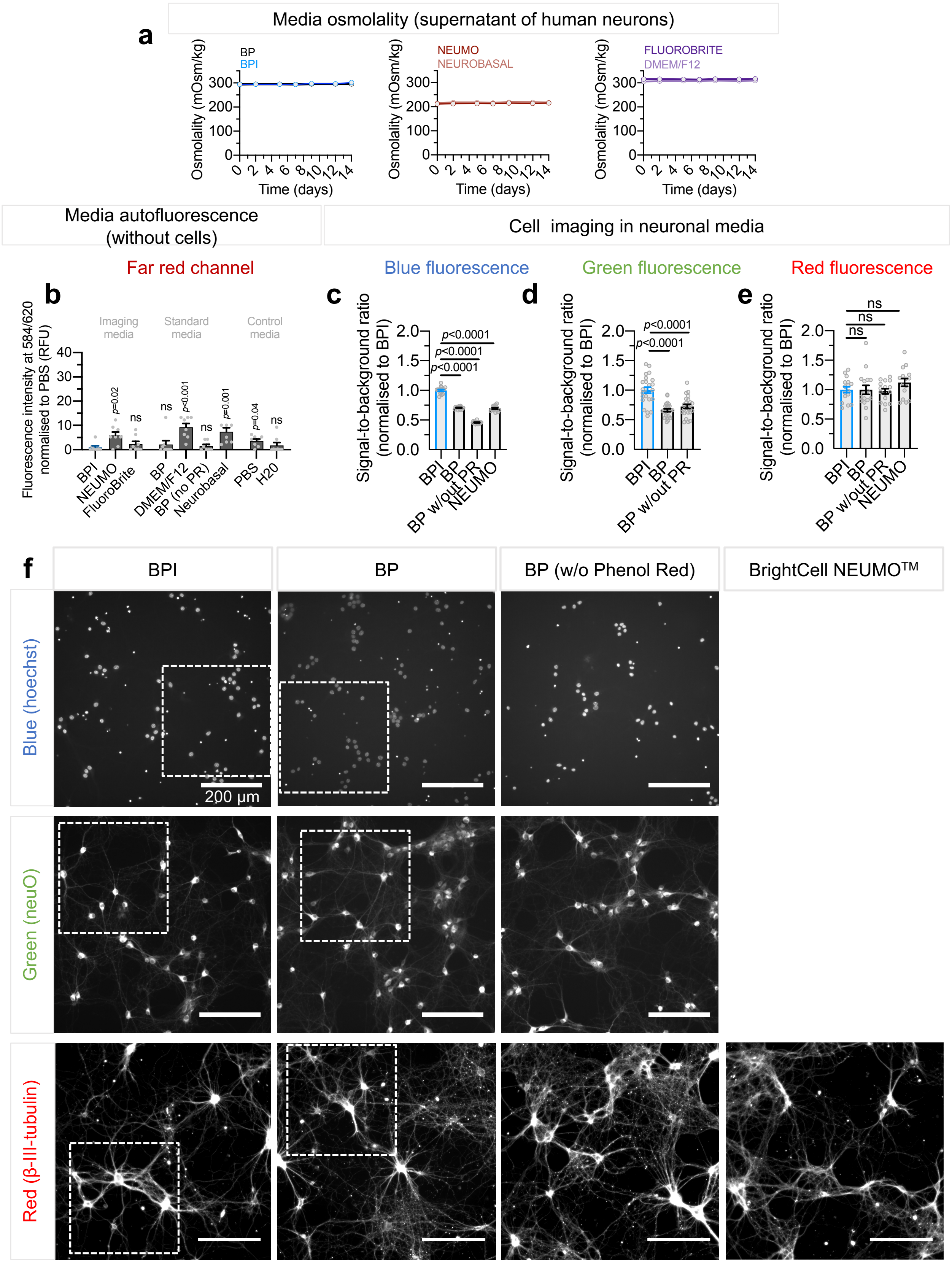

Supplementary Figure 1 (related to Figure 1 and 2). **Influence of BrainPhys Imaging on autofluorescence and signal-to-background ratio compared to competitive media. a** The osmolality of supernatant collected from human cortical iPSC-derived neurons matured in BP > 12 weeks and then switched to either BrainPhys Imaging (BPI), BrainPhys (BP), NEUMO, Neurobasal, DMEM/F12 or FluoroBrite basal media for 14 days (day 0: n = 3 per condition; day 2-14: n = 4 per condition). The same supplements for cortical neurons were added to all conditions. BPI maintained osmolality measurements over the 14 days at physiological levels. For each time point, results were generated from four replicate wells per medium. Far-red autofluorescence intensity of imaging, standard and control media collected in a 96-well plate reader set to an excitation/emission of 584/620 nm. Results were generated from eight replicate wells per medium (n = 8) analyzed across three independent experiments. For normalization, the mean fluorescence intensity in PBS was subtracted from the other media. **c-e** The signal-to-background ratio quantification of live rat cortical primary neurons labelled with NeuroFluor NeuO and fixed neurons stained with β-III-tubulin or Hoechst 33342 and imaged in BPI and BrainPhys (standard or without phenol red) and NEUMO. For BPI, the signal-to-background ratio of Hoechst 33342 **(c)** was significantly higher compared to BP, BP without Phenol Red and NEUMO. BPI also improved image quality relative to BP and BP without Phenol Red for neurons labelled with NeuO **(d)**. No significant differences were found for β-III-tubulin between conditions **(e)**. Across Hoechst 33342, NeuO and β-III-tubulin channels, a total of 16, 25 and 16 respective field-of-views were analyzed from one well per condition. Data were collected from two biologically independent experiments and normalised to BPI. **f** Example images of primary rat cortical neurons imaged in BPI, BP, BP without Phenol Red and NEUMO. Images were collected from two biologically independent experiments (Hoechst/NeuO; β-III-tubulin) with one well per condition. White boxes represent cropped full-scale images shown in Fig. 2e for BPI and BP conditions. Images are displayed with the following maximum/minimum intensity counts across all test media: 0/8500 (Hoechst), 0/3600 (NeuO), 1000/12000 (β-III-tubulin). Values in **(b-e)** represent mean ± SEM, significance determined via two-tailed non-parametric unpaired (Mann Whitney) tests. P-values are shown. ns, P > 0.05.

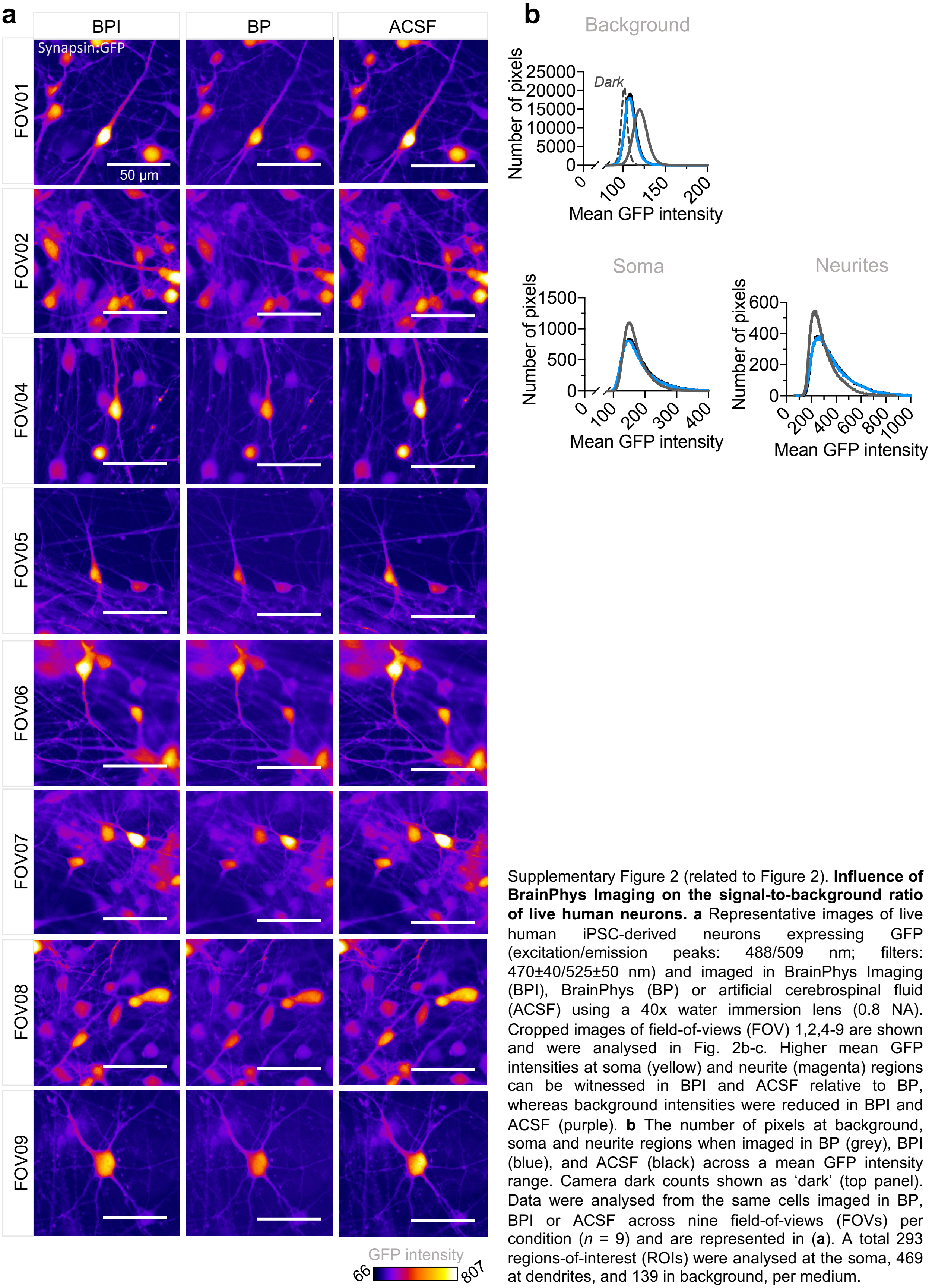

Supplementary Figure 2 (related to Figure 2). **Influence of BrainPhys Imaging on the signal-to-background ratio of live human neurons. a** Representative images of live human iPSC-derived neurons expressing GFP (excitation/emission peaks: 488/509 nm; filters: 470±40/525±50 nm) and imaged in BrainPhys Imaging (BPI), BrainPhys (BP) or artificial cerebrospinal fluid (ACSF) using a 40x water immersion lens (0.8 NA). Cropped images of field-of-views (FOV) 1,2,4-9 are shown and were analysed in Fig. 2b-c. Higher mean GFP intensities at soma (yellow) and neurite (magenta) regions can be witnessed in BPI and ACSF relative to BP, whereas background intensities were reduced in BPI and ACSF (purple). **b** The number of pixels at background, soma and neurite regions when imaged in BP (grey), BPI (blue), and ACSF (black) across a mean GFP intensity range. Camera dark counts shown as 'dark' (top panel). Data were analysed from the same cells imaged in BP, BPI or ACSF across nine field-of-views (FOVs) per condition (*n* = 9) and are represented in (**a**). A total 293 regions-of-interest (ROIs) were analysed at the soma, 469 at dendrites, and 139 in background, per medium.

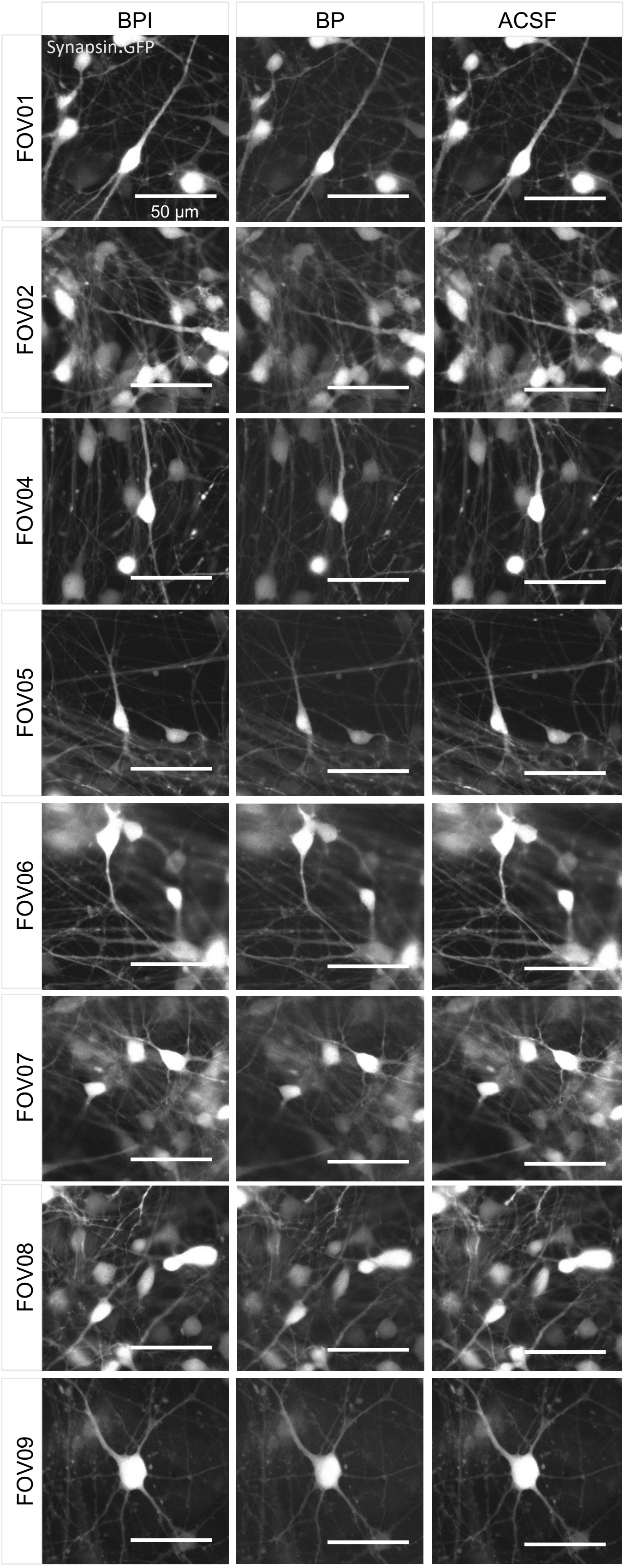

Supplementary Figure 3 (related to Figure 2). **Representative images of live human neurons in BrainPhys Imaging compared to ACSF and BrainPhys.** Representative gray-scale images of live human iPSC-derived neurons expressing GFP as shown in Supplementary Fig. 2a. Each image represents a single FOV in each condition, imaged across one coverslip. Images are displayed with maximum/minimum intensity counts of 75/400 across all test media. BPI, BrainPhys Imaging; BP, BrainPhys; ACSF, artificial cerebrospinal fluid.

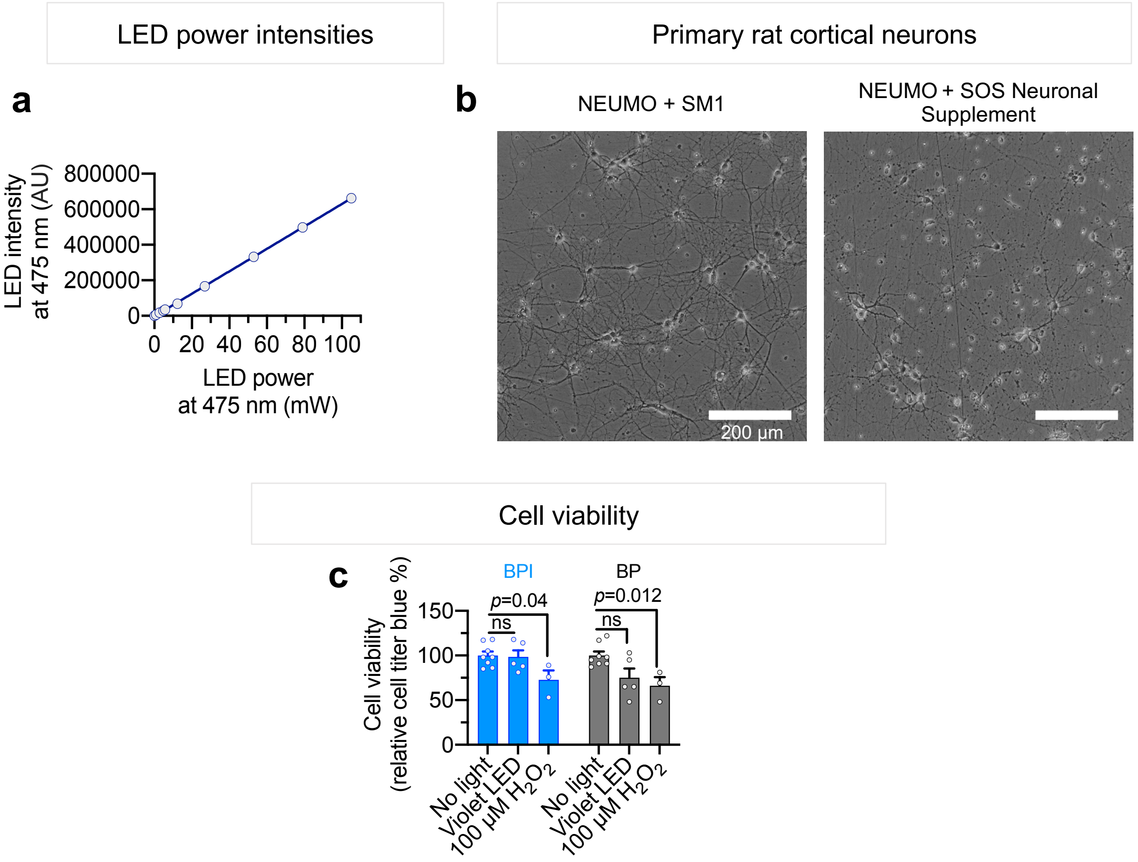

Supplementary Figure 4 (related to Figure 3). **BrainPhys Imaging maintains cell viability following light exposure.** **a** LED intensity recorded from a single LED on a LUMOS optical stimulator (at 475 nm), and its corresponding LED power (mW). **b** Primary rat cortical neurons could not be sustained for 11 days in vitro in NEUMO supplemented with SOS compared to NEUMO supplemented with SM1. Images are displayed from two replicate wells, collected across one independent experiment. **c** The cell viability (CellTiter-Blue® reagent) of primary rat cortical neurons was assessed following violet LED exposure inside a temperature-controlled incubator for 1 hour and treatment with 100 µM of H_2O_2. Data were collected across 3-5 independent experiments across three biological replicates. Results are shown normalised to 'no light' or 'control' conditions. Cell viability in BrainPhys Imaging (BPI) or BrainPhys (BP) media supplemented with SM1 following violet LED light exposure was maintained across all conditions but significantly reduced after H_2O_2 treatment in BPI and BP media. Values are presented as mean ± SEM. Significance determined in (**c**) via two-tailed non-parametric unpaired (Mann Whitney) test. ns, $P > 0.05$.

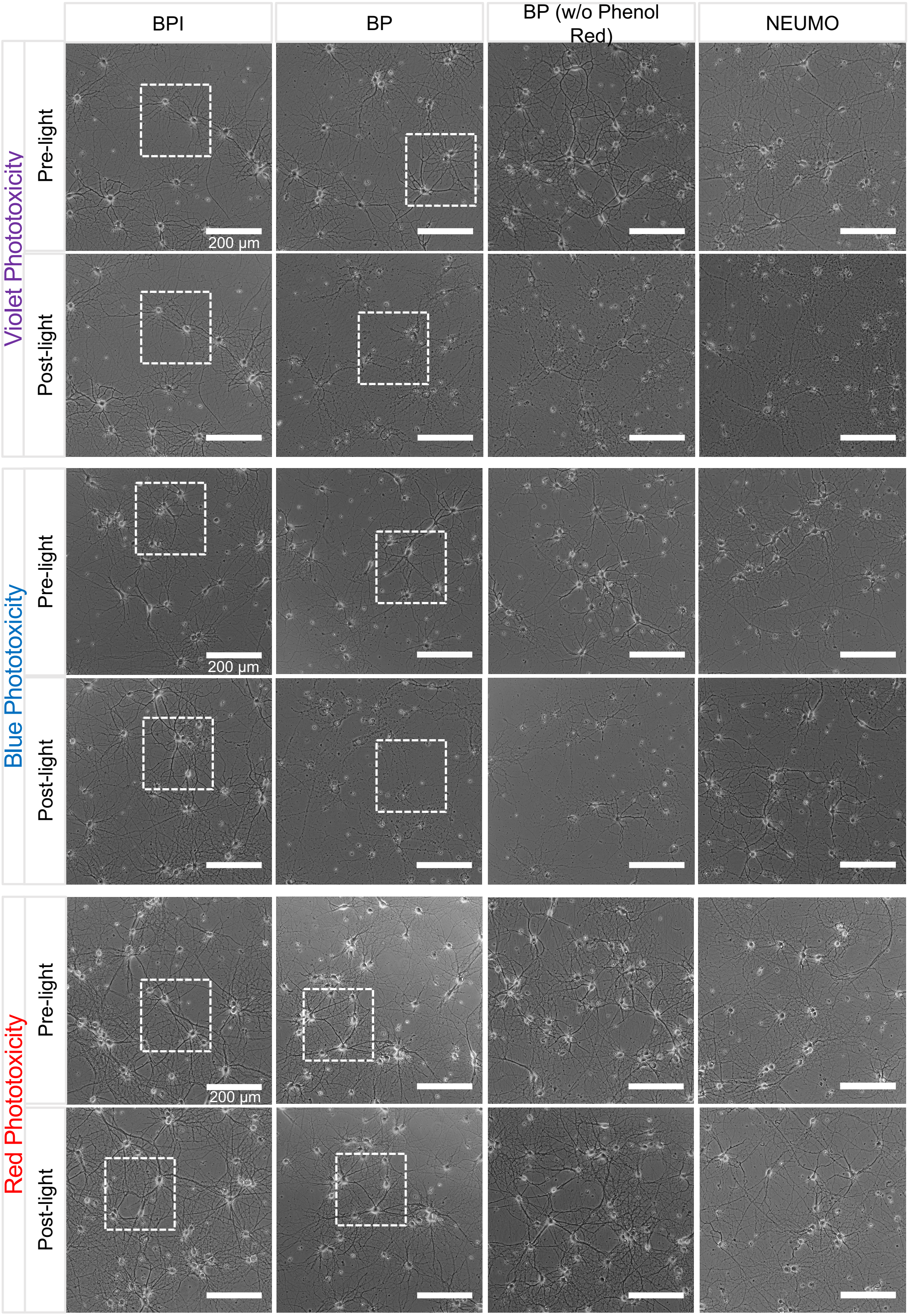

Supplementary Figure 5 (related to Figure 3). **BrainPhys Imaging reduced phototoxicity in neuronal cultures.** Full-scale representative images of rat cortical primary neurons cultured in standard BrainPhys (BP), BrainPhys Imaging (BPI), BrainPhys without Phenol Red (BP no PR) and NEUMO before and after exposure to red, blue and violet LED light. Images were collected from three independent experiments across three biological replicates per condition. Each image represents a single field-of-view (FOV) analysed in Fig. 3a-c. White boxes represent cropped images displayed in Fig. 3a-c.

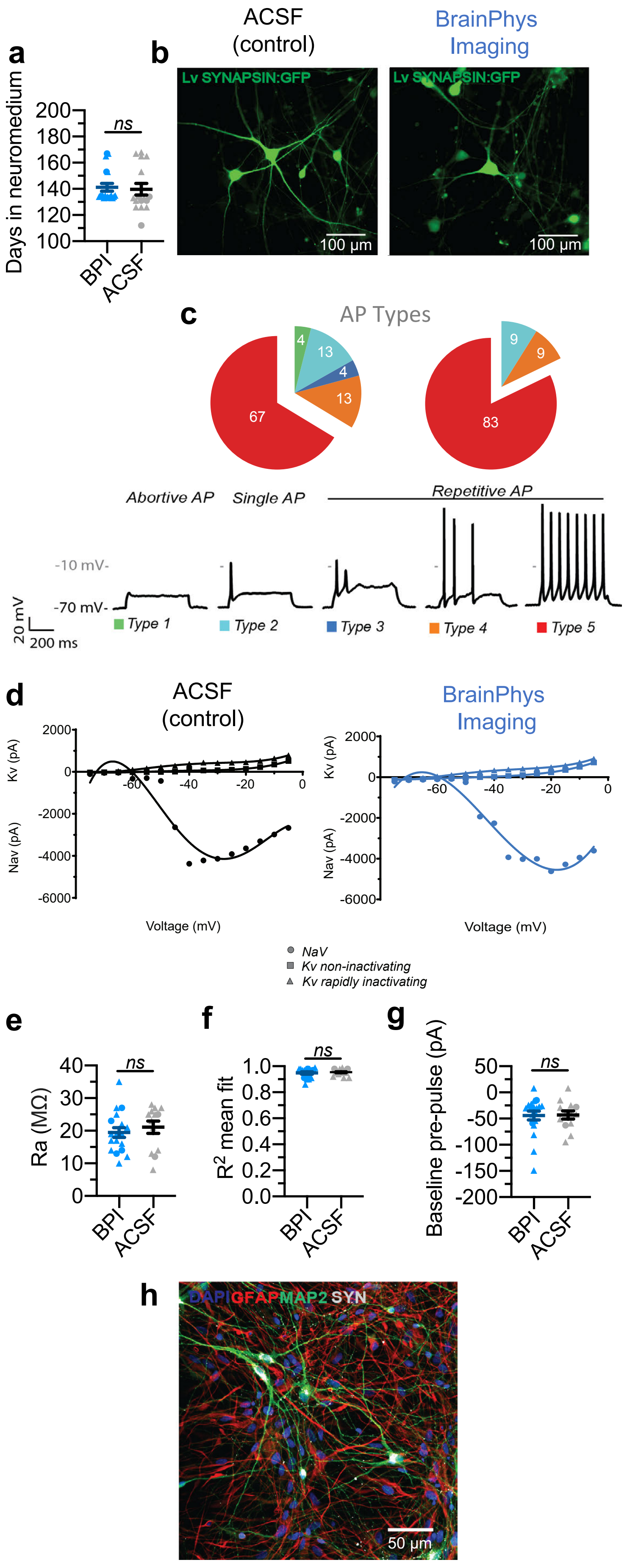

Supplementary Figure 6 (related to Figure 4). **Relative to ACSF, BrainPhys Imaging supports equivalent electrophysiological activity in human neurons. a** The maturation time (days) of Type 5 human neurons analysed and recorded across eighteen coverslips, incubated in BrainPhys + supplements ≥112 days (37 ºC, 5% CO_2 and 21% O_2) and then patched in BrainPhys Imaging (BPI; *n* = 19 neurons) or artificial cerebrospinal fluid (ACSF; *n* = 16 neurons). **b** Example images of neurons in ACSF or BPI expressing synapsin (GFP) before patch-clamping. **c** The proportion of action potential (AP) types evoked in human neurons when patched in BPI or ACSF with corresponding exemplary traces. Data were collected from a total of 47 neurons across eighteen coverslips in BPI (*n* = 23) or ACSF (*n* = 24). Type 5 neurons (evoked APs >10 Hz; >-10 mV peak amplitudes), Type 4 neurons (evoked APs <10 Hz, >-10mV peak amplitude), Type 3 neurons (>1 evoked AP and >1 aborted spikes < -10mV peak amplitudes), Type 2 neurons (>1 evoked AP >-10mV amplitude, followed by a plateau), Type 1 neurons (not able to fire APs >-10mV). **d** Analyzed current-voltage (IV) curves of selected Type 5 human neurons patched in ACSF or BPI medium, showing the relationship between voltage-dependent Na+ and K+ (non-inactivating and rapidly inactivating) currents (pA) and depolarizing current steps (mV). Voltage clamped at -70 mV with +5 mV steps. **e-g** Quantification of access resistance (Ra), baseline pre-pulse and R_2 mean fit, for all Type 5 neurons in BPI (*n* = 19) and ACSF (*n* = 13). Symbols in (**a,e-g**) represent human neurons tested first (triangles) or second (circle) in either medium. **h** Example immunostaining of human neuronal cultures matured in BrainPhys + supplements (>4 weeks). DAPI (blue), GFAP (red), MAP2 (green), Synapsin (grey) highlight neuronal and astrocyte populations along with dendritic projections. Values are presented as mean ± SEM. Significance in (**a,e-g**) determined via two-tailed non-parametric unpaired (Mann Whitney) test. ns, *P* > 0.05.

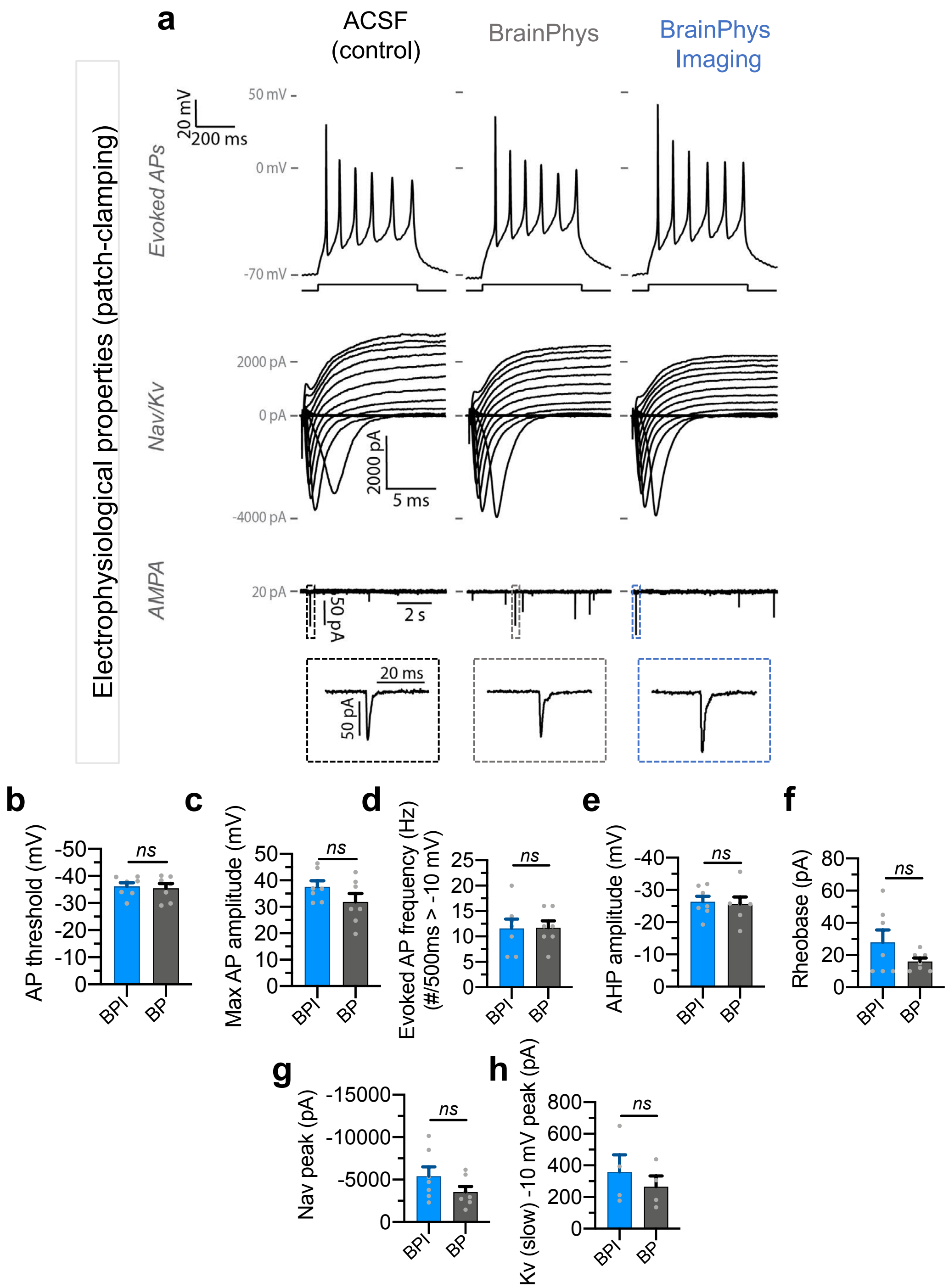

Supplementary Figure 7 (related to Figure 4). **Relative to BrainPhys, BrainPhys Imaging supports equivalent electrophysiological activity in human neurons.** Single-cell patch-clamp recordings of human pluripotent stem cell-derived neurons matured in standard BrainPhys (BP) + supplements medium for >12 weeks, and patch clamped in BP or BrainPhys Imaging (BPI) media. All patch-clamped cells (n = 8) included in the analysis were classified as Type 5 neurons (evoked APs >10 Hz, amplitudes >-10 mV) based on methods previously established in Bardy *et al.* 2016[1]. See also Fig. 4 and Supplementary Fig. 6. A subset of neurons (n = 7) was recorded in both media. All neurons were patched from a total of 4 coverslips. Each point on the graphs in panels (**b-h**) represents a single neuron. **a (top)** Shows similar evoked action potential (AP) traces following a 500 ms depolarizing current step for the same neuron patched in ACSF, BrainPhys and BPI. **a (middle)** Corresponding current-voltage characteristics (I-V curve) reveal similar voltage-dependent sodium (Nav) and potassium (Kv) current amplitudes for the same neuron patched across ACSF, BrainPhys or BPI. Nav and Kv current traces shown, respectively, below and above the *x*-axes. Current steps of +5 mV increments were used from resting potential at -70 mV. **a (bottom)** Typical spontaneous ePSC traces from the same neuron patch-clamped in ACSF, BrainPhys and BPI. No significant differences were found between the electrophysiological properties of human neurons patch-clamped in either BP (n = 7) or BPI (n = 7) basal media for (**b**) AP thresholds, (**c**) peak AP amplitudes, (**d**) firing frequencies of AP evoked by 500 ms depolarization steps (spikes with amplitudes >-10 mV included), (**e**) peak afterhyperpolarization (AHP) amplitudes, (**f**) rheobase values, and (**g**) peak Nav current amplitudes. **h** Peak amplitudes of slowly inactivating Kv currents in BPI (n = 4) and BrainPhys (n = 4) were also similar. Values are presented as mean ± SEM. Significance determined via two-tailed non-parametric unpaired (Mann Whitney) tests (**b-g**) and paired (Wilcoxon) tests (**h**). ns, P > 0.05.

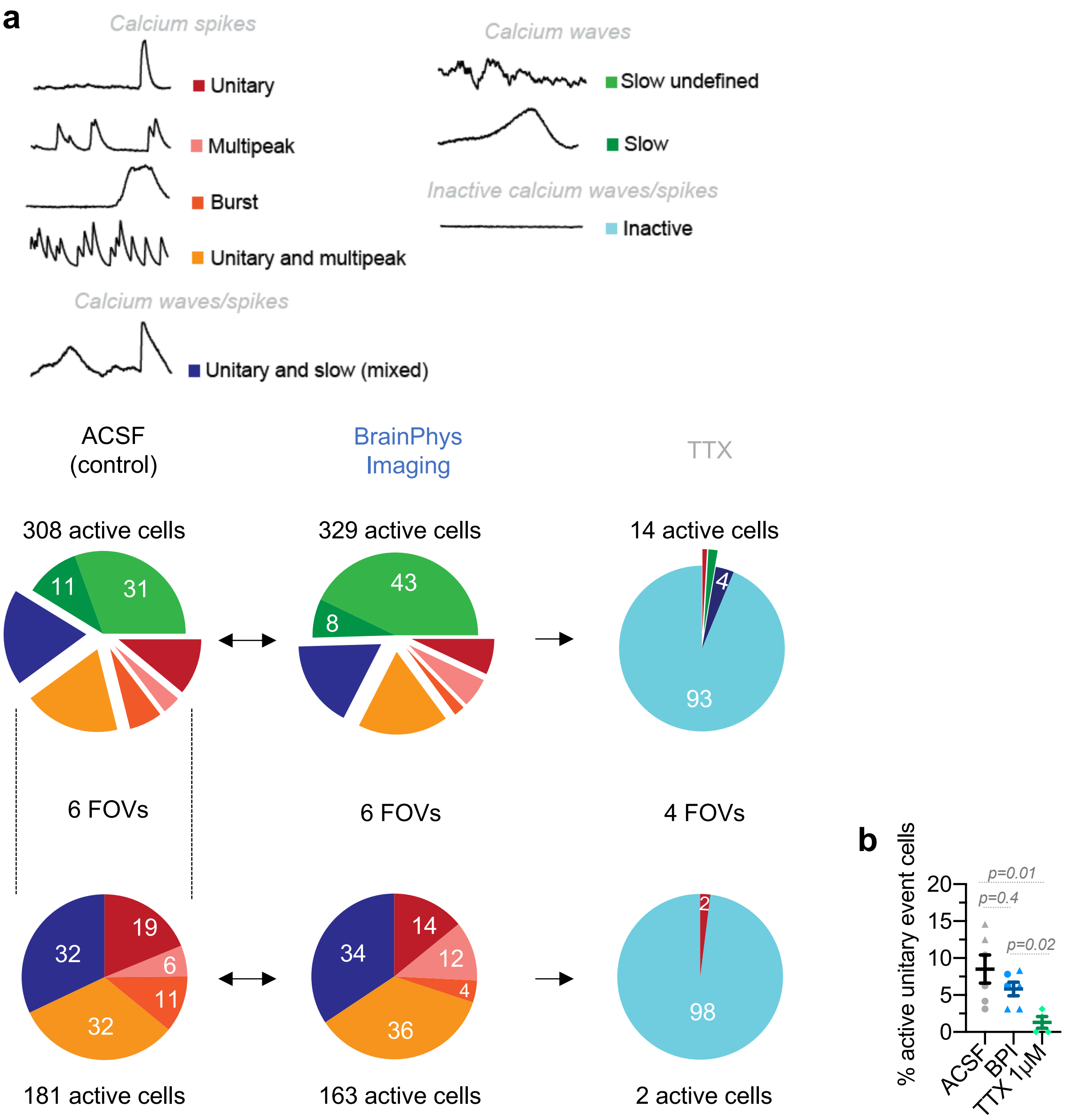

Supplementary Figure 8 (related to Figure 6). **Calcium imaging of human neurons in BrainPhys Imaging versus ACSF shows similar proportions of wave and spike events. a** Representative calcium image-sequence traces filmed at 5 Hz from human neurons in BPI or ACSF. Events were categorized into calcium spikes (fast rising phase events), calcium waves (slow rising phase events), calcium waves/spikes (a combination of fast and slow rising phase events) or inactive events. Top pie chart: calcium imaging data collected across up to six field-of-views (FOV) from two coverslips in ACSF (*n* = 308 cells), BPI (*n* = 329 cells) and TTX (*n* = 14 cells) were categorized into calcium event types. Bottom pie chart: a similar distribution of calcium spike event types was witnessed in selected cells initially displaying active calcium spikes in ACSF (*n* = 181 cells) then switched to BPI (*n* = 163 cells) and TTX (*n* = 2 cells) perfusates. TTX perfusion showed a significant reduction in active spike events. Note that cells inactive across all three perfusates were excluded from the analysis. **b** Quantification of the mean percentage of cells with unitary events witnessed per FOV in different perfusates. Symbols in (**b**) represent the order of media perfusion for each FOV: first (triangle), second (circle), or last (rotated square). Data is presented as mean ± SEM. Significance determined via two-tailed non-parametric unpaired (Mann Whitney) tests. *P*-values are displayed.

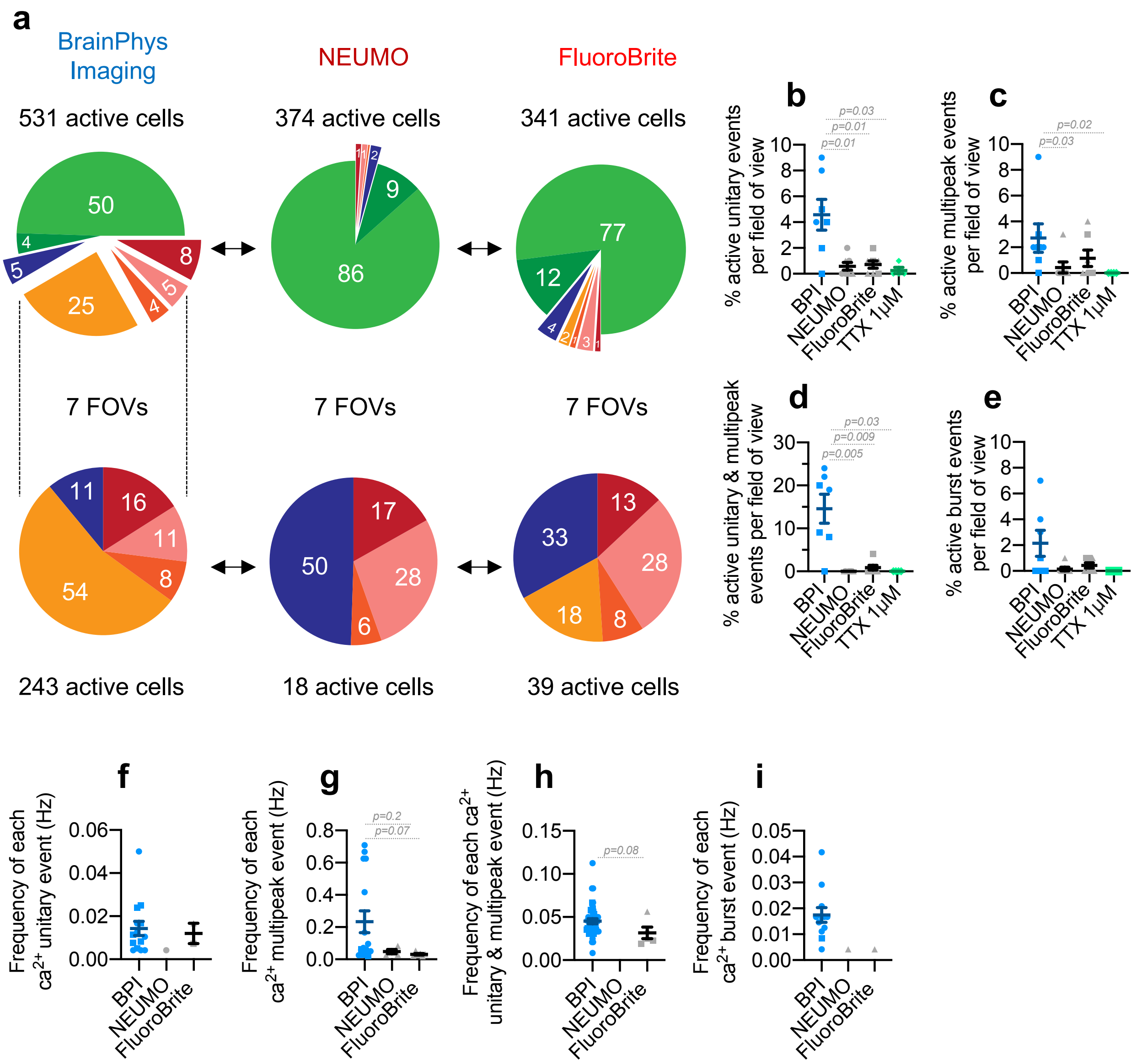

Supplementary Figure 9 (related to Figure 6). **Influence of BrainPhys Imaging medium on the activity of human neurons compared to BrightCell™ NEUMO and FluoroBrite™ DMEM. a (top)** Breakdown of time-lapse image sequences (filmed at 5 Hz, 1200 frames) measuring intracellular calcium events (Fluo-4 AM calcium sensor) categorized into active calcium spikes, waves, and spikes/waves. A total of 909 cells across 7 field-of-views (FOV) were recorded, and inactive cells across all three perfusates were excluded from further analysis. Remaining active cells in BPI (*n* = 531 cells), NEUMO (*n* = 374 cells) or FluoroBrite (*n* = 341 cells) perfusates across two coverslips were selected and analysed. **a (bottom)** Percentage breakdown of active calcium spikes and spike/wave events when imaged in either BPI (*n* = 243 cells), NEUMO (*n* = 18 cells) and FluoroBrite (*n* = 39 cells) perfusates. **b-i** Quantification of the mean percentage (**b-e**) and frequency (**f-i**) of active unitary, multipeak, unitary and multipeak, and burst events per FOV in the different media. Note that the addition of TTX (1 μM) perfusate completely blocked all unitary spike event activity. Symbols in (**b-i**) represent the order of media perfusion for each FOV: first (triangle), second (circle), third (square) or last (rotated square). Data in (**b-i**) are presented as mean ± SEM. Significance determined via two-tailed non-parametric unpaired (Mann Whitney) tests. *P*-values are displayed.

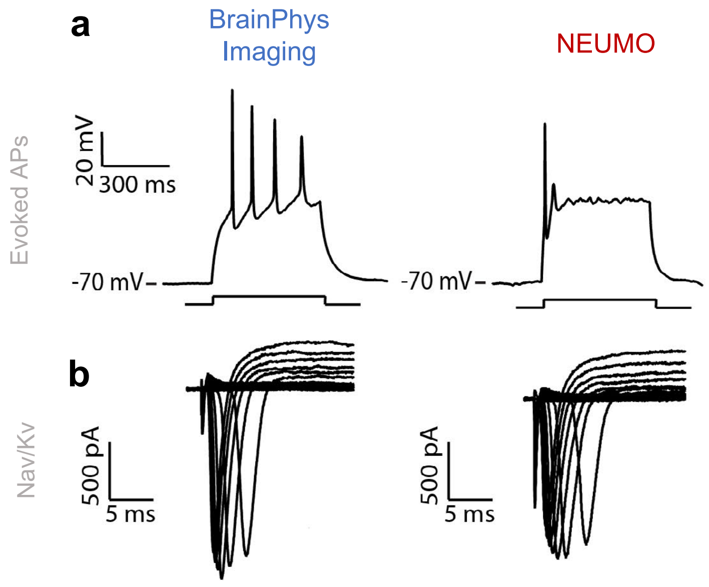

Supplementary Figure 10 (related to Figure 7). **BrightCell™ NEUMO limits the basic firing properties of human neurons compared to BrainPhys Imaging.** Example single-cell recording from a Type 4 human neuron patch-clamped in BPI or BrightCell™ NEUMO perfusate. **a** Typical evoked action potential (AP) traces following a 500 ms depolarising current step when patched and held at -70 mV in either perfusate. **b** Current-voltage characteristics (I-V curve) of a human neuron patched in either perfusate when held at −70 mV with +5 mV current steps. Voltage-dependent sodium (Nav) and potassium (Kv) current traces shown, respectively, below and above the *x*-axes.

Supplementary Table 1. Materials used in this study.

Antibodies	Source	Identifier
Chicken polyclonal anti-MAP2	Abcam	ab5392 (Lot No. GR3265288-3)
Donkey anti-chicken Alexa Fluor®488	Jackson Immunoresearch	703-545-155 (Lot No.116967)
Donkey anti-chicken Alexa Fluor®647	Jackson Immunoresearch	703-605-155 (Lot No.114163)
Donkey anti-mouse Alexa Fluor®488	Jackson Immunoresearch	715-545-150 (Lot No. 132977)
Donkey anti-rabbit Alexa Fluor®488	Jackson Immunoresearch	711-545-152 (Lot No. 116141)
Goat anti-mouse DyLight®594	Thermo Fisher Scientific	35510 (Lot No. M163715)
Mouse monoclonal anti-β-III-tubulin	BioLegend	801201 (Lot No. B264428)
Rabbit polyclonal anti-Synapsin1	Merck	ab1543 (Lot No. 2930537)
Chemicals, Peptides and Recombinant Proteins	Source	Identifier
Accutase	STEMCELL Technologies	07920
Anti-Adherence Rinsing Solution	STEMCELL Technologies	07010
Ascorbic Acid (Method #1)	Sigma	A4403
Ascorbic acid (Method #2)	STEMCELL Technologies	72132
BDNF (Method #1)	STEMCELL Technologies	78133.1
BDNF (Method #2)	STEMCELL Technologies	78005
BrainPhys Imaging™	STEMCELL Technologies	05796
BrainPhys™ Neuronal Medium	STEMCELL Technologies	05790
BrainPhys™ without Phenol Red	STEMCELL Technologies	05791
Brightcell™ NEUMO® Photostable Media	Merck	SCM146
BrightCell™ SOS® Neuronal Supplement	Merck	SCM147
CellTiter-Blue® Cell Viability Assay	Promega	G8081
CloneR™	STEMCELL Technologies	05888
CryoStor® CS10	STEMCELL Technologies	07930
CytoTox 96® Non-Radioactive Cytotoxicity Assay	Promega	G1780
Dibutyryl cAMP (Method #1)	Sigma	D0627
Dibutyryl-cAMP (Method #2)	STEMCELL Technologies	73882
Dispase	STEMCELL Technologies	07923
DMEM (high glucose, no glutamine)	Thermo Fisher Scientific	11960044
DMEM/F12 + GlutaMAX™ Basal Medium	Thermo Fisher Scientific	10565018
DMEM/F12 + HEPES Basal Medium	STEMCELL Technologies	36254
Donkey serum	Merck	S30
Fetal Bovine Serum, Qualified, Australia Origin	Thermo Fisher Scientific	10-099-141
FGF2 (bFGF)	STEMCELL Technologies	78134
FGF8b	PeproTech	100-25
Fluo-4, AM, cell permeant	Thermo Fisher Scientific	F14201
FluoroBrite™ DMEM	Thermo Fisher Scientific	A1896701
GDNF (Method #1)	STEMCELL Technologies	78139.1
GDNF (Method #2)	STEMCELL Technologies	78058
Gentle Cell Dissociation Reagent	STEMCELL Technologies	07174

GlutaMAX Supplement	Thermo Fisher Scientific	35050061
Hank's Balanced Salt Solution	Thermo Fisher Scientific	14025092
Hoechst 33342 (bisBenzimide H 33342 trihydrochloride)	Sigma	14533 (Lot No. 056M4091V)
Hydrogen Peroxide, 3% solution	Sigma	88597
IGF-I, Human Recombinant	STEMCELL Technologies	78022.1
IWP-2	Selleckchem	S7085
JC-1 Dye (Mitochondrial Membrane Potential Probe)	Thermo Fisher Scientific	T3168
Laminin (Method #1)	Thermo Fisher Scientific	23017015
Laminin (Method #2)	Sigma	L2020
LDN193189	STEMCELL Technologies	72147
Lenti-X™ qRT-PCR Titration Kit	Takara Bio	631235
L-Glutamic acid	Sigma	G8415
L-Glutamine	STEMCELL Technologies	07100
Matrigel, hESC-Qualified Matrix, LDEV-free	Corning	356277
mTeSR™1	STEMCELL Technologies	85850
N2 Supplement-A	STEMCELL Technologies	07152
Neurobasal™ Medium	Thermo Fisher Scientific	21103-049
NeuroCult™ Neuronal Plating Medium	STEMCELL Technologies	05713
NeuroCult™ SM1 Neuronal Supplement	STEMCELL Technologies	05711
NeuroCult™ SM1 Without Vitamin A	STEMCELL Technologies	05731
NeuroFluor™ NeuO	STEMCELL Technologies	01801
Noggin	PeproTech	120-10C
Papain	Worthington	LK003176
Paraformaldehyde, 4% in PBS	Alfa Aesar	J61899
Phosphate Buffer Solution (PBS)	STEMCELL Technologies	840020
Polyethylenimine (PEI), linear, 25 kDa	Polysciences	23966-1
Poly-D-lysine	Sigma	P7280
Poly-L-lysine hydrobromide	Sigma	P1524
Poly-L-ornithine hydrobromide (Method #1)	Sigma	P3655
Poly-L-ornithine, 0.01% solution (Method #2)	Sigma	P4957
Puromycin	Cayman Chemical	13884
ROS-Glo™ H_2O_2 Assay	Promega	G8820
SAG	Selleckchem	S7779
SB431542	STEMCELL Technologies	72232
Sodium Pyruvate, 100 mM solution	Sigma	S8636
Sonic Hedgehog (SHH)	PeproTech	100-45
STEMdiff™ Dorsal Organoid Kit	STEMCELL Technologies	08620
STEMdiff™ Neuron Differentiation Kit	STEMCELL Technologies	08500
STEMdiff™ Neural Organoid Supplement D	STEMCELL Technologies	08631
STEMdiff™ Ventral Organoid Kit	STEMCELL Technologies	08630
Tetrodotoxin	Abcam	ab120054

TWEEN® 20	Sigma	P7949
Y-27632	Cayman Chemical	10005583
Biological Samples	Source	Identifier
E18 rat cortex	BrainBits, LLC	SDECX
H9 (WA09) Human Embryonic Stem Cell Line	WiCell Research Institute	WA09 (Lot No. WB66595)
Lenti-X™ 293T Cell Line	Takara Bio	632180
Software	Source	Identifier
AxIS Aquisition Software (Version 2.4)	Axon Instruments	
Clampfit (v10.7)	Molecular Devices	
GloMax (v3.1)	Promega	
ImageJ (Version 1.51)	ImageJ	https://imagej.net/
MetaXpress (Version 6.2.2)	Molecular Devices	
Micro-manager	ImageJ	https://imagej.net/Micro-Manager
NeuralMetric (Version 2.5.1)	Axion Biosystems	
PClamp v10 (v10.7)	Molecular Devices	
Prism (v8.2.1)	GraphPad	https://www.graphpad.com/scientific-software/prism/
SoftMax Pro (Version 7.1)	Molecular Devices	
Imaging Resources	Source	Identifier
10x dry objective (NA 0.25)	Olympus	CACHN10XIPC
10x dry objective (NA 0.4)	Leica	HC PL APO CS2
10X water immersion lens (NA 0.3)	Olympus	UMPLFN10XW
20x dry objective (NA 0.4)	Nikon	Ph1 S Plan Fluor ELWD ADM
40X water immersion lens (0.8 NA)	Olympus	LUMPLFLNI40XW
63x oil objective (NA 1.4)	Leica	HC PL APO CS2
BX51 upright Microscope	Olympus	
CKX53 microscope	Olympus	
FITC/Cy2 filter Set	Chroma	49002
ImageXpress Micro 4 High Content Screening System	Molecular Devices	
PCO.Panda 4.2 Digital Camera	PCO	
pE300 (White)	CoolLED	
SP8 confocal microscope	Leica	
Electrophysiology Resources	Source	Identifier
Axon Digidata 1550B	Molecular Devices	
Master 9 programmable pulse stimulator (A.M.P.I)	Science Products	
Minipuls 3 (Peristaltic Pump)	Gilson	
Multiclamp 700B	Molecular Devices	
Tissue Culture Filters and Plates	Source	Identifier
37 µm reversible strainer	STEMCELL Technologies	27250
40 µm Cell Strainer, Falcon®	Thermo Fisher Scientific	352340
6-well suspension culture plate	STEMCELL Technologies	27145
96-well U-bottom plate	Corning	7007
AggreWell™ 800	STEMCELL Technologies	34811

Syringe filters cellulose acetate membrane (surfactant-free), membrane diam. 28 mm, pore size 0.45 µm	Corning	431220
Absorbance Recording Resources	Source	Identifier
CARY 7000 Spectrophotometer	Agilent Technologies	
Osmolality Resources	Source	Identifier
Disposable tubes (Osmometer)	Advanced Instruments	22-046733
Fiske Micro-Osmometer (Model 210)	Advanced Instruments	14-727-420
Probe Cleaner (Osmometer)	Advanced Instruments	3MA800
Multi-Electrode Array (MEA) Resources	Source	Identifier
48-Well Lumos MEA plate	Axion Biosystems	M768-tMEA-48OPT
Cytoview MEA 48 well plate	Axion Biosystems	M768-tMEA-48B
LUMOS Optical Stimulator	Axion Biosystems	
Maestro Pro MEA System	Axion Biosystems	
Autofluorescence Recording Resources	Source	Identifier
CellCarrier-96 Black, Optically Clear Bottom, 96-Well with Lid	Perkin Elmer	6005550
FLUOstar Omega (Multi-mode Plate Reader)	BMG Labtech	
MicroHR	Horiba	
Razoredge 405 nm longpass filters	Semrock	LP02-405RU-25
Razoredge 488 nm longpass filters	Semrock	LP02-488RE-25
Razoredge 532 nm longpass filters	Semrock	LP03-532RE-25
Toptica iCHROME MLE	Farmington	
Light Intensity Resources	Source	Identifier
PM100D Compact Power and Energy Meter Console	Thorlabs	
Phototoxicity Resources	Source	Identifier
58-watt NL-T8 Fluorescent Lamp Spectralux©Plus	Radium	
Blue LED lights	ER CHEN	ED0193L
Cellgard ES ClassII Biological Safety Cabinet	NuAire	S480-600E
Flame VIS-NIR spectrometer	Ocean Insight	
GloMax Microplate Reader	Promega	GM3000
Neon Transfection System	Thermo Fisher Scientific	
Red LED lights	ER CHEN	ED00021
SpectraMax M5 Multi-mode Microplate Reader	Molecular Devices	
Sper Scientific Model Light Meter	Sper Scientific	37350
Violet LED lights	AMARS	UV443528WP
Reagents for ACSF	Source	Identifier
Calcium chloride dihydrate ($CaCl_2 \cdot 2H_2O$)	Sigma	21097
Dextrose (D-(+)-Glucose)	Sigma	G7021
Magnesium sulfate heptahydrate ($MgSO_4 \cdot 7H_2O$)	Sigma	63138
Potassium chloride (KCl)	Sigma	60128

Sodium bicarbonate ($NaHCO_3$)	Sigma	S5761
Sodium chloride (NaCl)	Sigma	S6191
Sodium phosphate dibasic (Na_2HPO_4)	Sigma	S5136
Sodium phosphate monobasic (NaH_2PO_4)	Sigma	S5011

Supplementary References

1. Bardy, C. et al. Predicting the functional states of human iPSC-derived neurons with single-cell RNA-seq and electrophysiology. *Mol Psychiatry* **21**, 1573-1588 (2016).
