# Supplementary material for: BrainPhys neuronal medium optimized for imaging and optogenetics in vitro
Source: Nat Commun. 2020 Nov 3;11:5550. doi: 10.1038/s41467-020-19275-x (PMC7642238; doi:10.1038/s41467-020-19275-x)
Supplement: Supplementary file 3 — Description of Additional Supplementary Files [file 41467_2020_19275_MOESM3_ESM.docx]

**Description of supplementary files**

File Name: Supplementary Movie 1

Description: Supplementary Movie 1 (Related to Figure 6B-H; S8A-B). Calcium imaging movie showing a network of human iPSC-derived neuronal incubated with Ca2+ sensor (Fluo-4 AM) and recorded in BrainPhys Imaging (BPI) medium. Intracellular calcium levels correlate with fluctuations in fluorescence intensity. Time-lapse image sequences were recorded at 5Hz across 1200 frames for a total of 4 minutes within a region of 248 x 248 pixels. Image sequence file displayed has been sped up to 63 frames per second for representation purposes.’
