# Supplementary material for: BrainPhys neuronal medium optimized for imaging and optogenetics in vitro
Source: Nat Commun. 2020 Nov 3;11:5550. doi: 10.1038/s41467-020-19275-x (PMC7642238; doi:10.1038/s41467-020-19275-x)
Supplement: Supplementary file 4 — Reporting Summary [file 41467_2020_19275_MOESM4_ESM.pdf]

nature research

Corresponding author(s): Dr. Cedric Bardy

Last updated by author(s): Sep 29, 2020

Reporting Summary

Nature Research wishes to improve the reproducibility of the work that we publish. This form provides structure for consistency and transparency in reporting. For further information on Nature Research policies, see our Editorial Policies and the Editorial Policy Checklist.

Statistics

For all statistical analyses, confirm that the following items are present in the figure legend, table legend, main text, or Methods section.

n/a	Confirmed	
☐	☒	The exact sample size (*n*) for each experimental group/condition, given as a discrete number and unit of measurement
☐	☒	A statement on whether measurements were taken from distinct samples or whether the same sample was measured repeatedly
☐	☒	The statistical test(s) used AND whether they are one- or two-sided<br>*Only common tests should be described solely by name; describe more complex techniques in the Methods section.*
☐	☒	A description of all covariates tested
☐	☒	A description of any assumptions or corrections, such as tests of normality and adjustment for multiple comparisons
☐	☒	A full description of the statistical parameters including central tendency (e.g. means) or other basic estimates (e.g. regression coefficient) AND variation (e.g. standard deviation) or associated estimates of uncertainty (e.g. confidence intervals)
☐	☒	For null hypothesis testing, the test statistic (e.g. *F*, *t*, *r*) with confidence intervals, effect sizes, degrees of freedom and *P* value noted<br>*Give P values as exact values whenever suitable.*
☒	☐	For Bayesian analysis, information on the choice of priors and Markov chain Monte Carlo settings
☒	☐	For hierarchical and complex designs, identification of the appropriate level for tests and full reporting of outcomes
☒	☐	Estimates of effect sizes (e.g. Cohen's *d*, Pearson's *r*), indicating how they were calculated

Our web collection on statistics for biologists contains articles on many of the points above.

Software and code

Policy information about availability of computer code

Data collection	All patch-clamp and optogenetics during patch-clamping data was collected using PClamp software (version 10). Multi-Electrode Array (MEA) data was collected using version 2.4 and 2.5 AxIS acquisition software (Axion Biosystems). Image and calcium-imaging data was recorded using Micro-Manager software (v1.4.2). Confocal images were acquired using LasX software (Leica), ImageXpress images using MetaXpress 6.2.2 software (Molecular Devices) and Phase contrast images using CellSens (Olympus). Lactate Dehydrogenase (LDH) and H2O2 measurements were collected using GloMax v3.1 (Promega). JC-1 and CellTiter-Blue fluorescence were collected with SoftMax Pro v7.1 (Molecular Devices). Further details are outlined in the method section of the manuscript.
Data analysis	All patch-clamp data was analysed using Clampfit v10.7. Calcium-imaging recordings were analysed using a combination of Clampfit v10.3 and Microsoft Excel (v 16). MEA recordings were analysed with NeuralMetricTool version 2.5.1 software (Axion Biosystems) and MATLAB (R2020a). Image analysis was conducted using a combination of ImageJ (v 1.5) andMetaXpress 6.2.2 software (Molecular Devices). Graphs were generated in GraphPad Prism 8. Further details are outlined in the method section of the manuscript.

For manuscripts utilizing custom algorithms or software that are central to the research but not yet described in published literature, software must be made available to editors and reviewers. We strongly encourage code deposition in a community repository (e.g. GitHub). See the Nature Research guidelines for submitting code & software for further information.

Data

Policy information about availability of data

All manuscripts must include a data availability statement. This statement should provide the following information, where applicable:

- Accession codes, unique identifiers, or web links for publicly available datasets
- A list of figures that have associated raw data
- A description of any restrictions on data availability

The raw data that supports the figures of this study are freely available upon reasonable request to the corresponding author.

Field-specific reporting

Please select the one below that is the best fit for your research. If you are not sure, read the appropriate sections before making your selection.

☒ Life sciences ☐ Behavioural & social sciences ☐ Ecological, evolutionary & environmental sciences

For a reference copy of the document with all sections, see nature.com/documents/nr-reporting-summary-flat.pdf

Life sciences study design

All studies must disclose on these points even when the disclosure is negative.

Sample size	No sample-size calculations were performed. All sample sizes are described in detail within the figure legends. Sample sizes for patch-clamp, optogenetic, multi-electrode array (MEA) and calcium imaging measurements were based on previous studies (ref - 1, 28).
Data exclusions	Cells failing quality check were excluded from the patch-clamp and calcium-imaging data set, as described in the methods and legends. For quantification of primary rat neurons: Images where the neuron count was above 55 were excluded due to likelihood of glial cell contamination with high nuclei counts. Exclusion did not change the significance of data. This exclusion criteria was pre-determined.
Replication	All replication details are described in the manuscript.
Randomization	Patch clamping, optogenetic, calcium imaging and imaging experiments (Figures 2A-C; 4; 6; 7B-E; S7; S8; S9) were pseudo-randomized to alternate perfusions between media. Images collected using ImageXpress Micro4 High Content Screening system were acquired based on a pre-determined and consistently applied grid pattern. Remaining experimental groups were randomly assigned.
Blinding	The exact same unbiased computerized analysis protocols were applied to all conditions tested. Signal-to-background ratio analysis (Figures 2E-F; S1C-F) and media autofluorescence analysis testings (Figures 1C-F) were conducted blind to group allocation. Blinded group allocation of experimental groups was not deemed necessary for patch-clamping, calcium imaging, and optogenetic experiments due to pseudo-randomized media perfusion. Image analysis of GFP expressing neurons were not analysed blinded as the same region of interests (ROIs) were analysed across all conditions for each field-of-view (FOV).

Reporting for specific materials, systems and methods

We require information from authors about some types of materials, experimental systems and methods used in many studies. Here, indicate whether each material, system or method listed is relevant to your study. If you are not sure if a list item applies to your research, read the appropriate section before selecting a response.

Materials & experimental systems

n/a	Involved in the study	
☐	☒	Antibodies
☐	☒	Eukaryotic cell lines
☒	☐	Palaeontology and archaeology
☐	☒	Animals and other organisms
☒	☐	Human research participants
☒	☐	Clinical data
☒	☐	Dual use research of concern

Methods

n/a	Involved in the study	
☒	☐	ChIP-seq
☒	☐	Flow cytometry
☒	☐	MRI-based neuroimaging

Antibodies

Antibodies used	Mouse IgG2a monoclonal anti-β-III-tubulin (Biolegend; Cat#801201), Chicken polyclonal anti-MAP2 (Abcam; Cat#ab5392), Rabbit polyclonal anti-Synapsin I (Merck; Cat#ab1543), Donkey anti-mouse Alexa Fluor®488 (Jackson Immunoresearch; Cat#715-545-150), Donkey anti-rabbit Alexa Fluor®488 (Jackson Immunoresearch; Cat#711-545-152), Donkey anti-chicken Alexa Fluor®647 (Jackson Immunoresearch; Cat#703-605-155), Donkey anti-chicken Alexa Fluor®488 (Jackson Immunoresearch; Cat#703-545-155), Goat anti-mouse DyLight®594 (Thermo Fisher Scientific; Cat#35510)

Validation	β-III-tubulin antibody: Validated in-house using immunochemistry with rat cortical neurons and compared to previously validated β-III-tubulin antibody. Stated as validated for immunocytochemistry by manufacture with relevant citations.<br><br>MAP2 antibody: Validated by manufacturer for immunofluorescence and cited in many publications for this application and cell type (provided on manufacturer website). Validated in-house using immunocytochemistry with rat cortical neurons as a positive control (positive control recommended by manufacturer).<br><br>Synapsin I antibody: Quality assurance statement from manufacturer "Immunohistochemistry(paraffin): Synapsin representative staining pattern/morphology in rat hippocampal neurons. Tissue was pretreated with Citrate pH 6.0, antigen retrieval. A previous lot of this antibody was diluted to 1:1000, using IHC-Select® reagents used with HRP-DAB. Immunoreactivity is seen directly associated with terminal end of neuronal cell body." Immunocytochemistry listed as key application on manufacturer website with tested concentrations. Cited in publications for this application and cell types (rat and human origin cells).

Eukaryotic cell lines

Policy information about cell lines

Cell line source(s)	WA09 (H9) embryonic stem cells (WiCell), WA0l (Hl) embryonic stem cells (WiCell), XCL-1-derived neural stem cells (XCell Science), STiPS-M0Ol induced pluripotent stem cells (in-house from bone marrow mesenchymal cells), H14-derived neural stem cells (XCell Science), STiPS-B004 induced pluripotent stem cells (in-house from CD34+ cord blood cells)
Authentication	In-house cell lines authenticated by karotyping, morphology, immunocytochemistry and flow cytometry. Statement from WiCell on human pluripotent stem cell lines "WiCell uses a five passage assay to determine if test materials meet established standards for pluripotent stem cell culture. Clients may select human embryonic stem (ES) cells and/or induced pluripotent stem (iPS) cells for QC testing. Cultures are verified as karyotypically normal by G-band both prior to and following QC testing. All assays are performed in triplicate. Following completion of a QC assay, clients are provided full reports indicating cell expansion (through five passages) and differentiation status (Oct 3/4 and SSEA4 marker expression) of the resulting cultures." Statement from XCell Science on neural stem cells "Our comprehensive analysis include microarray analysis of gene expression, PCR, and immunocytochemistry as well as assessing differentiation into neurons and astrocytes."
Mycoplasma contamination	Mycoplasma contamination: In-house cell lines tested for mycoplasma contamination by PCR. Commercial cell lines were not tested for mycoplasma.
Commonly misidentified lines<br>(See ICLAC register)	None of the cell lines used are listed in the ICLAC register.

Animals and other organisms

Policy information about studies involving animals; ARRIVE guidelines recommended for reporting animal research

Laboratory animals	This study did not involve laboratory animals. E18 rat cortices were purchased from Brain Bits, LLC.
Wild animals	This study does not involve wild animals.
Field-collected samples	This study did not involve field-collected samples.
Ethics oversight	No ethical guidance or approval was required.

Note that full information on the approval of the study protocol must also be provided in the manuscript.
